# Pre- and Post-Surgical Nutrition for Preservation of Muscle Mass, Strength, and Functionality Following Orthopedic Surgery

**DOI:** 10.3390/nu13051675

**Published:** 2021-05-15

**Authors:** Katie R. Hirsch, Robert R. Wolfe, Arny A. Ferrando

**Affiliations:** Center for Translational Research in Aging & Longevity, Donald W. Reynolds Institute on Aging, Department of Geriatrics, University of Arkansas for Medical Sciences, Little Rock, AR 72205, USA; RWolfe2@uams.edu (R.R.W.); AFerrando@uams.edu (A.A.F.)

**Keywords:** atrophy, aging, anabolic resistance, essential amino acids, protein quality, dietary supplements, nutrient timing

## Abstract

Nutritional status is a strong predictor of postoperative outcomes and is recognized as an important component of surgical recovery programs. Adequate nutritional consumption is essential for addressing the surgical stress response and mitigating the loss of muscle mass, strength, and functionality. Especially in older patients, inadequate protein can lead to significant muscle atrophy, leading to a loss of independence and increased mortality risk. Current nutritional recommendations for surgery primarily focus on screening and prevention of malnutrition, pre-surgical fasting protocols, and combating post-surgical insulin resistance, while recommendations regarding macronutrient composition and timing around surgery are less established. The goal of this review is to highlight oral nutrition strategies that can be implemented leading up to and following major surgery to minimize atrophy and the resultant loss of functionality. The role of carbohydrate and especially protein/essential amino acids in combating the surgical stress cascade and supporting recovery are discussed. Practical considerations for nutrient timing to maximize oral nutritional intake, especially during the immediate pre- and post- surgical periods, are also be discussed.

## 1. Introduction

Nutritional status is a strong predictor of postoperative outcomes. Malnourished patients have longer lengths of stay, higher readmission rates, a greater number of complications, and higher mortality risks [1,2]. An estimated 24–65% of surgical patients, ranging from young adults to the elderly undergoing major surgery, are malnourished or at risk of malnutrition [3,4], a percentage that only increases over the course of a hospital stay [5]. Protein intake is especially important for modulating surgical stress and supporting recovery, yet surgical patients significantly under-consume protein, taking in about 22–36% of estimated requirements [6]. Consequences of malnutrition and inadequate protein intake are more serious for older adults, due in large part to the challenge of maintaining muscle mass. Malnutrition in older patients leads to rapid deterioration of cardiometabolic health, strength, functionality, independence, and an increased risk of mortality [7]. Nutrition is recognized as an important component of enhanced recovery after surgery (ERAS), which is designed to minimize stress and facilitate the return of functionality after surgery [1,8]. Currently, nutrition recommendations leading up to and following surgery primarily focus on screening and prevention of malnutrition. Recommendations regarding fasting and nutrient content and timing immediately before and after surgery are evolving [1,8,9]. Specific recommendations regarding macronutrient composition and timing around surgery, especially for protein, have not been established. The goal of this review is to highlight the role of carbohydrate and protein in supporting surgical stress and recovery. Practical considerations to maximize oral nutritional intake during the immediate pre- and post- surgical periods will be discussed, with emphasis on protein intake to minimize muscle atrophy and loss of functionality following orthopedic surgery.

## 2. The Surgical Cascade

For a patient in a non-stressed, clinical state, surgery stimulates a cascade of inflammatory, immune, and metabolic responses that result in a hypermetabolic-catabolic state [10]. Stimulated by the upregulation of glucagon, cortisol, and proinflammatory cytokines, significant catabolism of hepatic and muscle glycogen occurs to meet the energy demands of wound healing. Gluconeogenesis in the liver is also significantly upregulated, relying on lactate, amino acids, and glycerol as primary precursors [11]. The elevation in catabolic hormones also interferes with insulin secretion, preventing blood glucose clearance. This causes an insulin resistant, hyperglycemic state. Depending upon the degree of surgical invasiveness, this response can last for a few hours to several days, and in some cases up to 2–4 weeks [12]. Insulin resistance is a hallmark stress response, occurring in healthy individuals and exacerbated in patients with diabetes or preexisting hyperglycemia. If uncontrolled, post-surgical insulin resistance can impair immune function, as well as increase the risk of infection and/or mortality [13,14].

Protein catabolism is elevated during the stress response. Due to the hypercortisolemia that occurs with surgery, protein synthesis is reduced, and protein breakdown increases. The net result is an efflux of amino acids from skeletal muscle to provide amino acid precursors for gluconeogenesis, wound healing, and immune function [12]. When the muscle catabolism associated with the stress response to surgery is coupled with the general state of immobility that accompanies major surgery, significant skeletal muscle loss can occur. In healthy individuals, loss of muscle tissue begins to occur in as little as 48 h of inactivity, with significant loss within five days. In healthy young men, total thigh muscle volume decreased by 1.7% (0.1 kg) and 5.5% (0.3 kg) after two and seven days of disuse, respectively, with the greatest amount of atrophy occurring in the quadriceps [15]. In older individuals, the rate of post-surgical atrophy is higher compared to young adults. Following total knee arthroplasty, atrophy has been estimated to be about 1% per day in older adults (65 years) [16], with decreases of up to 18% in the quadriceps and hamstring of the surgical leg in six weeks. A majority of this atrophy (80%) occurred during the first two weeks post-surgery [16,17].

Loss of strength and functionality follows from muscle atrophy. Exercise, specifically resistance training, is the most effective way to prevent muscle atrophy. However, surgery often requires a period of total or partial immobility. After seven days of immobilization, a 5.5% decrease in thigh muscle volume was accompanied by significant decreases in leg extensor (−19%), leg press (−21%), and calf strength (−8%) in healthy young men [15]. In older adults, deterioration is even faster. Older adults have been shown to experience up to 14.3% decrease in muscle volume within two weeks following total knee arthroplasty [16], along with significant declines in mobility tests such as chair stand, stair climb, timed up-and-go, and six-minute walk [16,18]. The longer the period of immobility, the greater the deleterious consequences. Following abdominal surgery, functionality was not fully regained for two months post operation, while almost a third of hip arthroplasty patients continue to experience moderate to severe activity limitations up to five years post-surgery [19]. In cases where exercise is not safe or feasible, such as the early post-operative period, nutritional strategies must be considered to mitigate the loss of muscle mass.

This review will highlight oral nutritional strategies that patients could easily implement prior to and following major surgery to preserve muscle or minimize atrophy and the resultant loss of functionality. Benefits of carbohydrates and protein/essential amino acids in supporting and accelerating the healing process, mitigating muscle atrophy, and maximizing post-surgical functional outcomes will be discussed. Practical recommendations for nutrient source and timing throughout all phases of the surgical process (pre-, peri-, and post-surgical) will also be discussed. Where direct evidence in surgical patients is limited, applied data from sport and injury recovery has been applied. For further information on other nutritional considerations and strategies to facilitate surgical recovery and rehabilitation, such as micronutrient intake and dietary supplements, readers are directed to other reviews [20,21].

## 3. Surgical Role of Carbohydrate and Protein

Nutritional intake around the operative period is aimed at supporting increased nutritional needs during the hypermetabolic and inflammatory state, managing post-surgical insulin resistance, and reducing muscle atrophy. Initial strategies have emphasized carbohydrate/glucose intake, in efforts to reduce post-surgical insulin resistance and the rise in gluconeogenesis [22]. Compared to fasting conditions, pre-operative glucose intake (8–10 oz containing 50 g, 2 h before surgery) has been shown to reduce post-operative insulin resistance by up to 50% [23]. In addition, pre-operative glucose intake has been shown to positively impact lean body mass maintenance and muscle function [24]. Oral ingestion of 125 mg/mL of carbohydrate consumed the night before and up to 3 h before surgery was effective in maintaining whole-body protein balance 24 h after surgery [24]. It was also beneficial in reducing the loss of quadricep strength one-week post-surgery (−11% vs. −16%) [18], while also increasing the rate of return to pre-surgical strength values at one (−5% vs. −13%) and two months post-surgery (+4% vs. −0.6%) [18].

Although beneficial, carbohydrate intake alone is limited in its capacity to support the catabolic state of surgery. First, the body has a limited storage capacity for glucose (<24 h) [23]. Under normal fasting conditions in healthy, young adults, glycogen stores decrease in an almost linear fashion over the first 22 h [25]. As glycogen stores decrease, the rate of gluconeogenesis increases, accounting for about 50% (range: 46–81%) of glucose production within 12 h [25,26]. Applying this to a surgical patient, 6–12 h of pre-surgical fasting would mean that gluconeogenesis is significantly upregulated before surgery even begins. Once surgery begins and the stress state is initiated, the rate of glycogen depletion will generally accelerate. A second limitation of glucose is that it does not fully suppress gluconeogenesis during the stress state. Although a 50% reduction in insulin resistance is beneficial, uptake and oxidation of glucose is still impaired, and catabolism/oxidation of amino acids remains elevated. Third, post-surgical carbohydrate intake has little effect on suppression of insulin resistance or utilization of amino acids for gluconeogenesis [12,23,27]. In major colorectal surgery patients, immediate post-operative consumption of either a eucaloric or hypocaloric meal had no effects on insulin resistance, even when a pre-surgical drink was provided [28,29]. Finally, glucose does not directly support or mitigate the elevated need for amino acids during the post-surgical period. While pre-surgical glucose intake can help alleviate the rate at which protein catabolism occurs, amino acids are required to sustain the increase in protein turnover that occurs in stress states.

Some degree of post-surgical muscle loss is inevitable. When protein intake is inadequate, skeletal muscle serves as the primary source of essential amino acids needed to maintain whole-body protein synthesis. Thus, there is an increased need for dietary protein post-surgically, in order to meet the body’s elevated amino acid needs and reduce the risk of muscle catabolism. The benefits of increased dietary protein intake in countering the catabolic effects of illness, injury, disuse, and aging have been previously discussed [30,31]. Post-surgical amino acid supplementation has been shown to effectively reduce whole-body and muscle catabolism, stimulating a 40% increase in whole-body protein synthesis and 20% reduction in whole-body protein breakdown [32]. Twelve weeks of essential amino acid supplementation has been shown to reduce muscle atrophy and enhance functional outcomes in low physical functioning older adults [33]. Following total knee arthroplasty, essential amino acid supplementation reduced quadriceps and hamstring atrophy in the operated leg at two weeks and six weeks post-surgery compared to placebo [16]. Muscle atrophy in the non-surgical leg was also reduced [16,17]. Essential amino acid supplementation seems to be effective for maintaining muscle strength, even when some atrophy occurs. Two weeks post total knee arthroplasty, amino acid supplementation increased leg extensor strength (+6.5% vs. −15.5%) and reduced the loss of leg flexor strength (−27.3% vs. −59.1%) compared to supplementation with non-essential amino acids [16]. In hip and knee arthroplasty patients, greater hip function was recovered by 2 weeks post-surgery [34] while leg strength was significantly increased above pre-surgery values at 8 weeks post-surgery [35]. When considering functional outcomes, amino acid supplementation has been shown to maintain get up-and-go times and improve stair climb times [16], but have minimal impact on walking capacity [16,35,36,37,38]. In older adults with low physical function, 12 weeks of twice daily amino acid supplementation promoted significant improvements in walk distance, grip strength, and leg strength [33].

In order to provide the best surgical support, both carbohydrate and protein intake should be considered. Glucose and protein both stimulate insulin release, but differ in their effect on post meal changes in blood glucose, differences that can be leveraged to optimize post-surgical glycemic regulation [30]. Carbohydrates produce a rapid increase in blood glucose, leading to hyperglycemia. A biphasic increase in insulin results, leading to subsequent increases in glucose oxidation and glycogen synthesis, while inhibiting fatty acid oxidation [30]. This biphasic response is altered in situations of insulin resistance, such as type 2 diabetes or post-surgical insulin resistance. On the other hand, protein consumption increases blood amino acid concentrations, which in turn stimulates insulin and muscle protein synthesis. [12,30,31]. The insulin response to protein intake is not negatively impacted by insulin resistance [39]. As such, combined amino acid and glucose intake can mitigate loss of muscle loss and strength [31,40,41], especially when consumed prior to surgery [23]. Following surgery, a combination of free-form essential amino acids plus dietary protein may be advantageous; stimulating a large, rapid increase in essential amino acids to activate protein synthesis, followed by a more prolonged availability of both essential and non-essential amino acids to maximize the net gain in body protein and gluconeogenic precursors [42,43]. Depending on proximity to surgery (weeks, days, hours before and after), different sources of carbohydrate and protein can be leveraged to maximize nutritional intake.

## 4. Nutrient Timing

Similar to an athletic endeavor, nutrient timing strategies can be implemented during both the pre- and post-operative periods to prepare the body for the stress of surgery, support increased metabolic demands, and offset the catabolic consequences. The goal of pre-operative nutrition is to ensure adequate energy stores to meet the demands of the stress state. The goal of post-operative nutrition, on the other hand, is to promote nitrogen balance, reduce the loss of lean mass, and facilitate rapid healing and recovery. Substantial developments in dietary and sport supplements provide significant opportunity to maximize nutritional intake during the immediate hours before and after surgery and should be given consideration for pre- and post-surgical nutrition. Oral nutritional supplements have also been shown to reduce the net cost associated with hospitalization by an average of 12.2%, an average of about $180 in orthopedic patients. Cost savings are associated with reduced complications (−35%) and length of stay (−2 days).

## 5. Pre-Operative Nutrition

**Figure 1:**
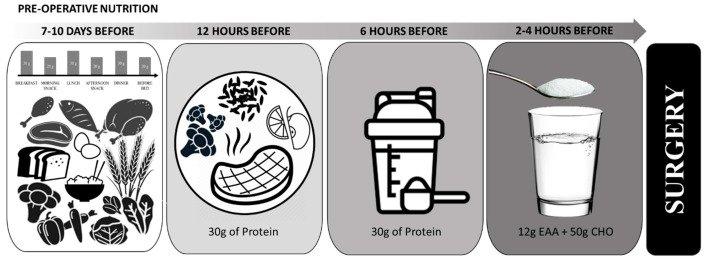
Example of pre-operative nutrient timing leading up to surgery. Modified from Smith-Ryan et al. (2020).

Starting 7–10 days prior to surgery (Figure 1), nutrition emphasizing both high-quality carbohydrate and protein intake would ensure optimal nourishment prior to surgery [8]. Preoperative carbohydrate loading is highly recommended for surgical patients [22]. Current carbohydrate loading recommendations typically focus on the evening before surgery [8,12]. However, it may be of greater benefit if initiated days prior to surgery (7–10 days). Using sports nutrition as a model, ingesting ~60% of total energy or 8 g per kg body mass per day of carbohydrate for a minimum of 3–4 days is effective for maximizing glycogen stores [44]. Emphasis on complex carbohydrates, such as vegetables, fruits, and whole grains, will also ensure adequate intakes of vitamins, minerals, and fiber, which are important in supporting immunity and the microbiome, which may have implications for nutrient absorption, reducing inflammation, and muscle recovery.

**Figure 2:**
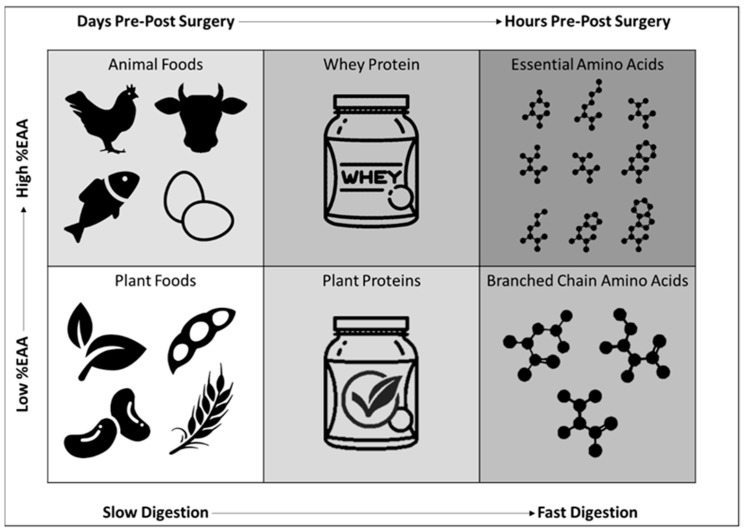
Protein sources with consideration of food source, protein quality, absorption, and EAA availability.

Protein intakes of 1.2–2.0 g/kg/day from high-quality protein sources distributed throughout the day (20–40 g of protein per sitting) is recommended by ERAS protocols to help ensure protein needs are met leading up to surgery [1,8,9,20]. Protein quality is based on four primary factors: (1) percentage of protein as essential amino acids (EAA), (2) profile of EAAs, (3) total ileal digestibility (TID), and (4) EAA bioavailability [45]. The highest quality source of protein would optimize each of these categories; providing a high percentage of EAAs, a profile of EAAs that matches amino acid requirements, and a high TID, resulting in an adequate delivery of EAA to appropriate tissue (Figure 2) [46]. Maximizing protein quality is especially important in overcoming anabolic resistance, defined as a reduction in the protein synthetic response to protein intake that occurs with aging and immobilization [47]. In terms of whole-food sources of protein, animal products, such as chicken, beef, fish, eggs, and milk, are considered the highest-quality. Compared to plant products, animal products contain a higher percentage of EAAs and leucine in amounts that correspond with the body’s requirements for growth, optimal health, and function [48]. Animal sources of protein also have higher bioavailability and are more easily digested than many plant products. Thus, when compared gram for gram, animal sources of protein stimulate a greater anabolic response compared to plant sources [49]. Although considered lower-quality, plant sources do contribute to overall protein intake. If consumed in higher quantities (1.2–1.6 g/kg/day) and from a variety of plant sources (complementary sources) to ensure adequate intake of all EAAs, plant sources of protein can meet protein needs in healthy adults [50,51]. Higher-quality plant sources of protein include lentils, quinoa, black beans, soy, peas, and rice [48]. As part of the natural food matrix, whole-food sources of protein (both animal and plant) are also rich in essential micronutrients, including vitamins, minerals, omega-3 fatty acids, fiber, antioxidants, and other phytonutrients that are important for overall health and recovery. Plant products are a rich source of vitamins, minerals, fiber, and secondary compounds, such as phenols, antioxidants, and other phytochemicals. Similarly, animal products provide essential nutrients such as zinc, selenium, iron, phosphorus, calcium, and vitamins B_12_ and D, compounds that are not abundantly available from plants [52].

Special attention should be given to nutritional intake in the 24 h period prior to surgery (Figure 1). Historically, strict fasting procedures have restricted nutritional intake in the hours before surgery due to concerns of regurgitation or aspiration under anesthesia [21]. As understanding of gastrointestinal motility, the harmful effects of long fasting periods on metabolic stress, insulin sensitivity, and recovery have improved, pre-surgical fasting recommendations have been revised. According to ERAS protocols, it is currently recommended that patients fast for 6 h following a light meal, and for 2 h from consumption of clear liquids prior to surgery [1,8,9]. While consuming whole-food sources of carbohydrate and protein is customary and advisable, their utility within in the 6-h pre-operative window is limited. Dietary supplements provide nutrition in a concentrated form, that is often more easily digested, absorbed, and a more effective form of nutrient delivery, making them ideal for the hours leading up to surgery. In compliance with current pre-surgical fasting recommendations, 6–12 h before surgery, patients should consume a well-rounded meal emphasizing complex carbohydrates and high-quality protein. For a morning surgery, consuming a quality dinner the evening before should be encouraged. For surgeries occurring later in the day, consuming a light breakfast in addition to a quality dinner the evening before may be recommended. Within 6 h of surgery, patients should begin abstaining from whole foods, but can continue to consume protein and carbohydrate containing beverages, such as a protein shake, a sports drink, or chocolate milk. Modified carbohydrate supplements, such as hydrothermally modified starches, may provide a unique benefit during this time period [53]. These complex carbohydrates were originally developed to sustain overnight glucose levels in children with glycogen storage disease. More recently, they have been utilized by endurance athletes to sustain glucose levels throughout long duration events. These modified starches are rapidly emptied from the stomach into the intestines, where they are slowly digested. Unlike maltodextrin ingestion, which results in a rapid increase in plasma glucose followed by hypoglycemia within 1.5 h post ingestion, modified starches have a lower glycemic response and sustain plasma glucose levels for up to at least 3.5 h post ingestion [53,54]. Sustained plasma glucose levels may be advantageous for offsetting the surgical stress response.

The most common forms of protein supplements include whey protein isolate and concentrate, casein, soy, and other plant-blends. Whey protein isolate is considered one of the highest quality protein sources, with more than 50% of the amino acid concentration coming from EAA and 2.7g of leucine in a common 25g dose [48,55]. Compared to whey concentrate, isolate has a greater concentration of protein per dose and reduces the amount of lactose, making it more tolerable for individuals with lactose intolerance. Similar to whey, casein protein is milk based and contains a high percentage of EAA (~48%). Casein is more slowly digested than whey, resulting in a slower EAA rate of appearance in the blood. Though not ideal for maximizing muscle protein synthesis, the prolonged anabolic response may be effective in reducing protein breakdown during long periods of fasting, such as leading into surgery [56] or the overnight period [57]. Soy is considered one of the highest quality plant sources of protein, making it one of the most common plant-based protein supplements. Soy protein is 38% EAA, making it less efficient compared to whey protein when matched for total grams of protein [48,55]. This can be overcome by consuming a greater quantity of protein (40g) and/or consuming a blend of plant-based proteins, a strategy intended to improve the EAA concentration and profile [58,59].

Two hours before surgery, patients are advised to start fasting from clear liquids. The ingestion of ~50g of carbohydrate two hours before surgery has been shown to preserve post-surgery insulin sensitivity with no increased risk of adverse events [60]. Since modified carbohydrate supplements rapidly empty from the stomach, consumption may sustain glucose levels for the duration of surgery. To our knowledge, the safety and efficacy of modified starches on mitigating the post-surgical stress response have not been tested. However, the mechanism of action is consistent with the amelioration of metabolic responses to surgery.

Co-ingestion of free form amino acids two hours before surgery should also be recommended to promote a positive protein balance prior to surgery. Since free form amino acids do not require cleaving of peptide bonds, they are rapidly absorbed into the blood stream. Free form amino acid supplements include EAAs (containing all nine essential amino acids), branched-chain amino acids (BCAA; containing three essential amino acids), and leucine (a single essential amino acid). Leucine is responsible for activating the anabolic response. However, without an adequate intake of the other EAAs, protein synthesis/turnover is substantially limited, as recently reviewed [61]. Similarly, the BCAAs, which include leucine, isoleucine, and valine, do not effectively support protein synthesis due to the lack of the other six EAAs [61]. Although the anabolic impact of BCAAs is limited, BCAAs supplementation can function as an alternative fuel source, reducing oxidation of endogenous amino acids during the hypermetabolic state [62]. In contrast to BCAAs, free form EAA supplements, containing all nine EAAs, have been shown to stimulate a greater anabolic response than whey or whole-food sources [63]. By elevating plasma leucine and other EAA levels to a greater degree than that from dietary protein digestion [46], free-form EAAs are effective in overcoming anabolic resistance when consumed alone or in combination with other protein sources [42,63]. Since free form EAA dissolve easily in water and have quick transit from the stomach, they represent an ideal supplemental option before surgery [17].

## 6. Post-Operative Nutrition

**Figure 3:**
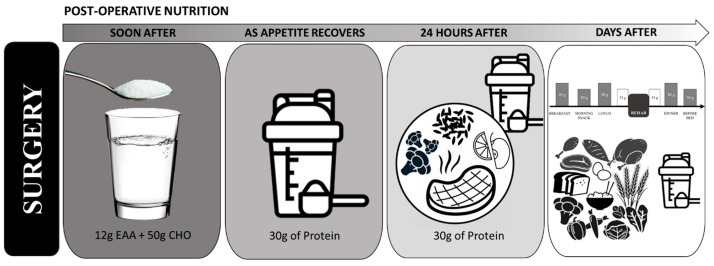
Example of post-operative nutrient timing. Partially adapted from Smith-Ryan et al. (2020).

Earlier post-operative feeding is associated with reduced infection complications, improved healing, and decreased length of stay. Thus, it is generally recommended to resume oral feeding as soon as possible post-surgery, with the goal of returning to solid foods within 24 h. Simply put, an effective approach to post-operative nutrition is pre-operative nutrition in reverse (Figure 3).

In the early hours post-surgery, patient appetite is often suppressed making consumption of solid foods difficult. During this time, encouraging consumption of free form EAAs would be advantageous. Free form EAAs have minimal effect on appetite [63] and do not interfere with the anabolic response of a subsequently consumed meals. The anabolic effects of free form EAAs are independent of insulin and not impaired by hypercortisolemia [31]. Although effects of EAA in the immediate post-surgical period have not been evaluated, the consumption of free form EAA as soon as possible post-surgery would be highly advantageous for providing the amino acids needed to support healing and recovery, without concern of a large gastric load. Amino acid intake in the immediate post-operative period could also help support the immune response [64]. As appetite is regained, patients can transition to protein beverages with greater calorie density, such as protein shakes, until able to consume whole food sources or meals. Consuming EAAs with whey protein and/or dietary protein may be especially advantageous throughout the post-surgical period, especially for older individuals.

During the rehabilitation period, protein intakes of at least 1.6 g/kg/day and up to 2.0–3.0 g/kg/day is generally recommended [20,65]. Similar to the preoperative period, this amount should be consumed throughout the day, 20–40 g of protein per sitting. However, appetite is often reduced following surgery, making it difficult to meet dietary protein goals. Consumption of EAAs and/or protein supplements between meals would be advantageous in achieving higher protein intake levels and optimize nutritional intake throughout the day when appetite is suppressed [20]. For surgeries that require physical rehabilitation, such as a joint replacement, nutrient timing around rehabilitation sessions should also be considered. Nutrient intake around an exercise/rehabilitation session can help increase lean mass, strength, and functionality, ultimately leading to faster return to activities of daily living [33,66,67]. Adequate nutrient intake before the rehabilitation session ensures energy and nutrients are available to maximize exercise performance, while nutrient intake following the session supports recovery and adaptation [20]. Starting 3–4 h before a therapy session, patients should consume a small meal, containing complex carbohydrates (50–100 g) and quality protein (30–40 g). Then, 15–45 min prior to therapy, a carbohydrate and protein containing beverage should be consumed, followed by another protein beverage after the therapy session [20]. In addition to protein, supplements such as creatine monohydrate, β-hydroxy-β-methylbutyrate (HMB), omega-3 fatty acid, and probiotic supplementation have demonstrated efficacy in the support of muscle, strength, and functionality [20,21], and may merit consideration.

## 7. Conclusions

Adequate nutritional consumption is essential for addressing the surgical stress response and mitigating the loss of muscle mass, strength, and functionality. Carbohydrate intake supports the elevated energy needs associated with post-surgical metabolic alterations and wound healing, while protein intake provides the amino acids required to support wound healing, immune function, and muscle preservation. Emphasizing protein intake throughout the entire surgical process, but especially in the post-surgical period, reduces muscle catabolism and the resultant loss of functionality. While high-quality, whole-food sources of carbohydrate and protein should be maintained, supplemental sources allow for continued nutrient intake within the hours immediately before and after surgery. Free-form EAAs, alone or in addition to other protein sources, are especially effective for overcoming anabolic resistance, warranting their use both before and after surgery to attenuate muscle atrophy in older adults. Considerations provided within this review are meant to compliment, not replace current peri-surgical recommendations, as outlined by governing organizations, and patients should always consult with a physician/nutritionist team for individualized recommendations [8].

## Data Availability

Not applicable.
